# Adipocyte-conditioned medium induces resistance of breast cancer cells to lapatinib

**DOI:** 10.1186/s40360-020-00436-z

**Published:** 2020-08-14

**Authors:** A. Geneste, M. N. Duong, L. Molina, L. Conilh, S. Beaumel, A. Cleret, K. Chettab, M. Lachat, L. P. Jordheim, E. L. Matera, C. Dumontet

**Affiliations:** 1 Centre de Recherche en Cancérologie de Lyon (CRCL), INSERM UMR 1052, CNRS 5286, 8 Avenue Rockefeller, 69008 Lyon, France; 2 Department of Oncology, Lausanne University Hospital Center (CHUV) and University of Lausanne, Epalinges, Switzerland; 3 Hospices Civils de Lyon, Banque de tissus et cellules, 5 place d’Arsonval, 69003 Lyon, France; 4 Hospices Civils de Lyon, Services d’Hématologie, 165 Chemin du Grand Revoyet, 69310 Pierre-Bénite, France

**Keywords:** HER2, Breast cancer, Resistance, Adipocytes, Lapatinib

## Abstract

**Background:**

The existence of a cross-talk between peritumoral adipocytes and cancer cells has been increasingly investigated. Several studies have shown that these adipocytes protect tumor cells from the effect of anticancer agents.

**Methods:**

To investigate a potential protective effect of adipocyte-conditioned medium on HER2 positive breast cancer cells exposed to tyrosine kinase inhibitors (TKI) such as lapatinib, we analyzed the sensitivity of HER2 positive breast cancer models in vitro and in vivo on SCID mice in the presence or absence of adipocytes or adipocyte-conditioned medium.

**Results:**

Conditioned medium from differentiated adipocytes reduced the in vitro sensitivity of the HER2+ cell lines BT474 and SKBR3 to TKI. Particularly, conditioned medium abrogated P27 induction in tumor cells by lapatinib but this was observed only when conditioned medium was present during exposure to lapatinib. In addition, resistance was induced with adipocytes derived from murine NIH3T3 or human hMAD cells but not with fibroblasts or preadipocytes. In vivo studies demonstrated that the contact of the tumors with adipose tissue reduced sensitivity to lapatinib. Soluble factors involved in this resistance were found to be thermolabile. Pharmacological modulation of lipolysis in adipocytes during preparation of conditioned media showed that various lipolysis inhibitors abolished the protective effect of conditioned media on tumor cells, suggesting a role for adipocyte lipolysis in the induction of resistance of tumor cells to TKI.

**Conclusions:**

Overall, our results suggest that contact of tumor cells with proximal adipose tissue induces resistance to anti HER2 small molecule inhibitors through the production of soluble thermolabile factors, and that this effect can be abrogated using lipolysis inhibitors.

## Background

Approximately 20% of human breast cancers possess an amplification of human epidermal growth factor receptor 2 (HER2), and this overexpression was previously correlated with aggressive tumor behavior and poor patient outcomes [1]. HER2 (ErbB2, or HER2/neu) is a member of the HER tyrosine kinase receptor (TKR) family, which includes three other members: the epidermal growth factor receptor (EGFR or HER1), HER3, and HER4. Homo- and hetero-dimerization of ligand-bound HER receptors results in activation of multiple pathways, including the p44/42 mitogen-activated protein kinase (MAPK) and phosphatidylinositol 3-kinase (PI3K)/AKT pathways, which regulate cell proliferation and apoptosis [2–4]. HER2 is the preferred heterodimerization partner of the other HER receptors, although it does not have a known natural ligand. Its activation is mediated by homodimerization, or by ligand-mediated stimulation of another HER receptor through heterodimerization. Targeted therapies such as monoclonal antibodies or tyrosine kinase inhibitors (TKI) have provided a significant improvement in patient outcome by targeting HER2 or its dimerization partners. The monoclonal antibody trastuzumab and the TKI lapatinib have both been approved by the FDA and the EMA in combination with chemotherapy [5, 6], and both have proven efficacy in the clinical setting [7, 8]. However, resistance still occurs in patients because of the complexity, robustness and redundancy of the HER biological network [9, 10].

Obesity has been described as a risk factor not only for cancer development but also for a decreased sensitivity to cancer treatment [11–14]. Epidemiological studies have demonstrated that trastuzumab and lapatinib were less efficient in overweight and obese patients than in other patients in terms of free relapse survival and overall survival [10, 15]. Indeed, with a 10-year follow-up the overall survival of lean women treated with trastuzumab was 38% higher than for obese women. Moreover, several in vitro studies have demonstrated a decrease of efficacy of targeted therapies on breast tumor cells when cells are cultured in the presence of adipocytes or adipocyte-conditioned medium (CM) [16–19]. The main mechanisms identified for obesity-mediated resistance of cancer cells to anticancer agents have been described in vitro or in mice and include alterations of the intracellular signaling pathway PI3K/AKT/mTOR [16], inhibition of cell cycle blockade and inhibition of apoptosis [20–22]. In a previous study, our team showed that adipocyte-secreted factors decreased the efficacy of trastuzumab on BT-474 and SKBR-3 breast cancer cell lines [16]. More recently we have shown that MVP could be involved in adipocyte-induced resistance of breast cancer cells to doxorubicin [23].

Several mechanisms of resistance have been described specifically for anti-HER2 targeted therapies. These include i) the expression of a truncated form of HER2 called p95HER2 unable to bind trastuzumab [24] ii) an alteration in ADCC mechanisms [25] iii) a defect in cell cycle arrest and/or apoptosis [26] iv) an alteration in phosphorylation of intracellular signaling pathways [27] v) the action of alternative tyrosine kinase protein activation in case of HER2 blockade [17, 28] vi) drug efflux through efflux pumps such as P-gp [29] and vii) an upregulation of estrogen receptors (ER) [30].

In this study, we analyzed the role of proximal adipose tissue in the resistance of breast cancer cells to small molecule targeted therapies such as the TKI lapatinib. Lapatinib is a small molecule that binds to the intracellular domain of the TKR and inhibits the activation of downstream signalization pathways. Lapatinib shares some of the mechanisms of resistance described for anti-HER2 targeted therapies. However, since it binds to the intracellular domain of HER2, the truncation of HER2 into p95HER2 and alterations in ADCC mechanism should not modify its activity, we did not study these mechanisms as a potential mechanism of resistance and focused our analyses on cell cycle arrest. Indeed, the cytotoxic effect of lapatinib has been described to modify the activation of the PTEN/AKT/mTor pathway and to block the cell cycle in G1 phase [31]. Several proteins are required for cell cycle progression, such as P27, AKT, cyclin D1 and E2F3. Phosphorylated AKT is involved in cell cycle progression by phosphorylating P27, thereby preventing cell cycle blockade. The non-phosphorylated form of P27 inhibits the action of cyclins, while E2F3 is implicated in cyclin D1 gene transcription.

After demonstrating the reduction of cell cycle blockade induced by lapatinib in the presence of adipocyte-conditioned medium, we reproduced the protective effect of adipocyte-secreted factors on tumor cells against lapatinib, according to studies carried on other compounds [16, 20, 32–34] and investigated the effect of adipocyte-conditioned medium on cell proliferation. In order to investigate whether the protective effect of adipocyte-secreted factors is dependent on HER2 expression, we explored the lapatinib-induced cytotoxic effect on different breast cancer cell lines in the presence or absence of adipocyte-conditioned medium. We reproduced these results in vivo in SCID mice using patient derived normal human adipose tissue. To understand the mechanisms of resistance to lapatinib induced by the proximity of adipocytes and to identify the different agents involved in these mechanisms, we performed different physical and chemical treatments on the adipocyte-conditioned media. In parallel, we investigated alterations occurring on breast tumor cells following the contact with adipocyte-conditioned media and the exposure to lapatinib, both at transcriptional and protein levels. Finally, we used pharmacological agents to modify the metabolism of adipocytes in order to determine the possibility to overcome adipocyte-induced resistance of tumor cells to lapatinib.

## Methods

### Cell culture

The preadipocyte cell line 3T3, the fibroblast cell line NIH3T3 and the tumor cell lines MDA-MB-453, MDA-MB-361, MDA-MB-231, and MCF-7 were cultured in complete DMEM medium (Life technologies), supplemented with 10% fetal calf serum (FCS), 2 mM L-glutamine, 100 U/mL penicillin and 100 μg/mL streptomycin). The tumor cell linesBT-474 and SKBR3 were cultured in complete RPMI medium with the same supplementation as DMEM. All cells were cultivated at 37 °C in presence of 5% CO_2_.

To induce the differentiation of 3T3 cells, confluent cells were incubated in differentiation medium (DMEM supplemented with 10% FCS plus 50 nM insulin (Sigma, 259,278)) up to 14 days. During the two-week incubation, pre-adipocytes differentiated into adipocytes (80–90% of the cells are differentiated after 14 days) and accumulate lipid droplets in their cytoplasm. The CM from pre-adipocytes (3T3-CM) and adipocytes (#3T3-CM) were harvested, centrifuged at 300 g for 5 min and either directly used or stored at − 20 °C before use. The control medium for these CM is complete DMEM (medium) for 3T3-CM and differentiation medium (#medium) for #3T3-CM.

#3T3 cells were incubated with 5 μM salbutamol, 18 μM terbutaline, 0,1 μM isoprenaline, 29,6 μM dobutamine, 45 μM propranolol, 45 μM atenolol, 15 μM insulin, 100 μM acipimox and 20 μM etomoxir.

### Protein denaturation, exosome isolation and lipid sequestration

The #3T3-CM was heated at 96 °C during 1 h in order to denature the proteins.

Exosomes were isolated from the CM by differential centrifugation. In brief, the CM was centrifuged at 3000 g for 30 min to remove cell debris then at 10,000 g for 60 min at 4 °C to separate vesicles from exosomes. The exosomes and the soluble factors were separated by ultracentrifugation at 100,000 g for 90 min. We evaluated the purification using the Nanosight® device that detects and quantifies the exosomes with a laser at 405 nm.

### Cytotoxicity MTT assay

BT-474, SKBR3 and MCF-7 cells were seeded in 96 wells plates at 20,000; 8000; 3000, cells per well respectively, in 50 μL media. The 3T3-CM, #3T3-CM, human adipocyte line hMAD-CM (hMADs-CM) and human fibroblast NIH3T3-CM (NIH3T3-CM) were added to the wells. The next day, drugs were added to the wells at a range of concentrations and cells were incubated for 72 h at 37 °C, 5% CO_2_. MTT was then added in each well and incubated for an additional 4 h. The supernatant was discarded, and a solution composed of isopropanol/H_2_O/HCl (90/9/1, v/v/v) was added. The optical density was determined at 540 nm using Multiskan device.

### Cell cycle analysis

BT-474 cells seeded in 6 well plates at 420,000 cells per well in 1.5 mL were incubated with 1.5 mL of either #3T3-CM or #medium or either 3T3-CM or medium, reproducing the MTT assays conditions for the indicated times. Each condition was performed in triplicate in at least three separate experiments, with or without lapatinib at 1 μM. Cells were harvested by trypsination and dead cells were numbered in the supernatant. After washing with PBS, the cells were incubated with propidium iodide solution (0.05 μg/mL) in the dark at 4 °C for 30 min. Cell cycle was measured by flow cytometry using a BD LSRII flow cytometer using BD FACSDiva software (BD Biosciences).

### Reverse transcription and quantitative PCR

BT-474 cells were exposed to #3T3-CM or 3T3-CM for 24 h prior to exposure to lapatinib. Cells were harvested, and RNA was extracted using a RNeasy Mini Kit (Qiagene®). To determine the impact of pharmacological agents on #3T3 cells, these were exposed for 6 h prior to RNA extraction using successively Qiazol® and formaldehyde.

Real-time qPCR was performed on a LightCycler Nano (Roche), detecting SYBR Green I signal transmission. The expression of genes was normalized to the housekeeping gene ribosomal 28S. Relative expression was determined using the ΔCt method.

### Patient samples

The body mass index (BMI) was calculated as follows: weight (kg)/height^2^ (m^2^). Subcutaneous adipose tissue obtained by lipoaspirate was immediately transported to the registered Cell Therapy Unit (Hospices Civils de Lyon, ETI/16/M/001). Briefly, after centrifugation (1962 g for 3 min), oil (upper phase) and tumescent phase (lower phase) were removed. Then, adipose tissue was digested with collagenase (0.1 U/ml, NB6 collagenase (GMP-grade, Serva Electrophoresis Roche, Indianapolis, USA) at 37 °C for 45 min and under constant shaking. Digestion was stopped by adding Dulbecco’s Modified Eagle’s Medium (DMEM with glutamax, Gibco (Invitrogen, Carlsbad, USA) containing 10% fetal calf serum (FCS, HyClone, Logan, USA). After centrifugation at 300 g for 5 min, floating adipocytes were discarded and cells from the stromal-vascular fraction (SVF) were pelleted, rinsed with medium, and centrifuged (300 g for 5 min at 20 °C). Cells were counted using 0.4% trypan blue (hospital pharmacy preparation) and viability was evaluated. Adipocytes were resuspended in DMEM for 24 h and supernatants were extracted and frozen at − 80 °C before use. Patient provided written informed consent.

### In vivo studies

All animal procedures were performed in accordance with the European Union directive 86/609/EEC. Experiments were performed under individual permit and in animal care facilities accredited by the French Ministry of Agriculture. The study was approved by the local animal ethics committee (University Claude Bernard Lyon I, protocol number DR-2014-64). The study was conducted using severe combined immunodeficiency (SCID) mice, with five mice per group, purchased from Charles River. Mice bearing xenografts of normal human adipose tissue were obtained by the subcutaneous injection of 1 mL of abdominal adipose tissue obtained from patients undergoing plastic surgery who had provided informed consent. After 1 week, BT-474 tumors were grafted subcutaneously in contact with the adipose xenograft. Treatments were initiated when the tumor volume was 150 mm^3^, with daily orally administration of lapatinib 135 mg/kg for 2 weeks. Tumor growth was directly measured using a caliper. The volume was calculated using the following formula V = 4/3 x π x R^3^ where R is the radius measured. Each mouse was euthanized after experimentation with carbon dioxide.

### Statistical analysis

Means ± SD or representative experiments are shown when experiments were repeated several times (*n* ≥ 3). Statistical significance was evaluated using paired Student’s *t*-test on the means of at least three independent in vitro experiments. Unpaired Student’s *t*-test was used for in vivo experiment. IC50 were calculated using Compusyn software. *p*-values below 0.05 (*) or 0.01 (**) were considered significant while “ns” stands for not significant data.

### Materials

Human breast cancer cell lines BT-474, SKBR3, MDA-MB-453, MDA-MB-361, MDA-MB-231, MCF-7 and the fibroblast cell line NIH3T3 were all obtained from ATCC. The 3 T3-F442A (named 3 T3 cells here) murine preadipocyte cell line was kindly provided by Dr. Catherine Muller (Toulouse, France). The human multipotent adipose-derived stem cell (hMADS) were provided by Dr. Christian Dani, *UMR 6543 CNRS,* Nice, France and cultured as described previously [16].

Lapatinib was purchased from Sigma Aldrich while phenylephrine, clonidine, epinephrine, dobutamine, yohimbine, propranolol and atenolol and ibrutinib were purchased from BioScience. Acipimox and etomoxir were obtained from Adooq Bioscience and terbutaline, prazosin, salbutamol, afatinib and AZD4547 were purchased from Selleckchem.

The primers used for PCR were: AKT forward primer 5′-tctggcttcatcggcagt-3′, AKT reverse primer 5′-gatcgcactccctgtctagg-3′, cycline D1 forward primer 5′-tacaaccaggcagcggata-3′, cycline D1 reverse primer 5′–agccacccagaattagacacc-3′, P27 forward primer 5′-ccctagagggcaagtacgagt-3′, P27 reverse primer 5′-agtagaactcgggcaagctg-3′, E2F3 forward primer 5′-acgaagtccagatagtccaaaaa-3′, E2F3 reverse primer 5′-ataccccatcgggtgactg-3′, FABP4 forward primer 5′-ggatggaaagtcgaccacaa-3′, FABP4 reverse primer 5′-tggaagtcacgcctttcata-3′, LPL forward primer 5′-tttgtgaaatgccatgacaag-3′, LPL reverse primer 5′-cagatgctttcttctcttgtttgt-3′, HIF1α forward primer 5′-catgatggctccctttttca-3′ and HIF1α reverse primer 5′-gtcacctggttgctgcaata-3′.

## Results

### Adipocyte-conditioned medium reduces lapatinib-induced cell cycle blockade in tumor cells

**Fig. 1:**
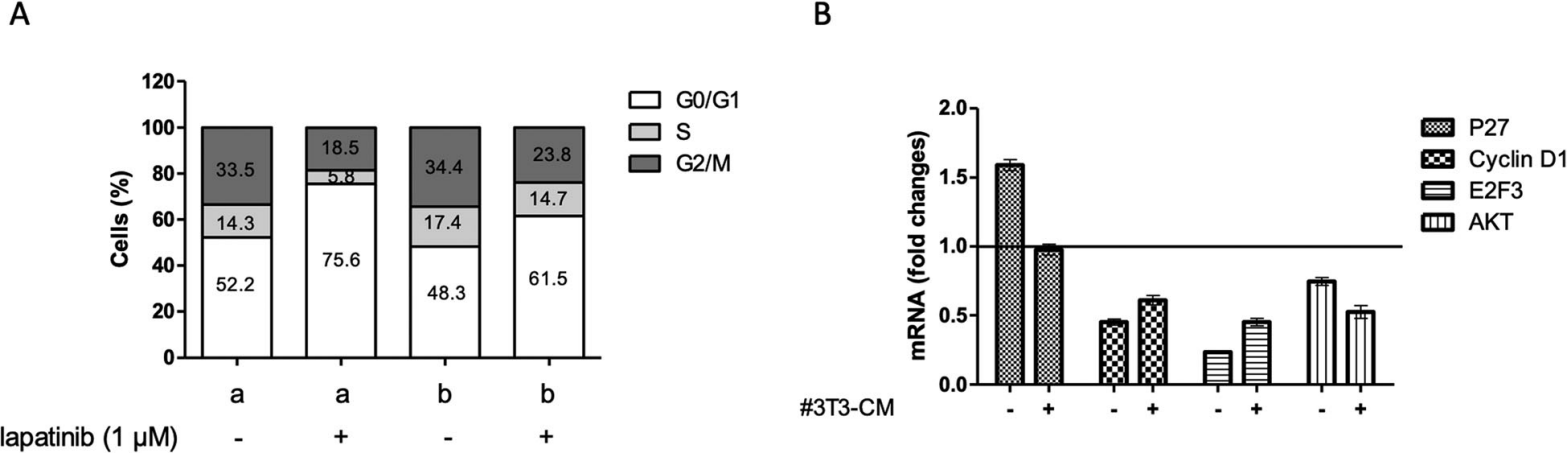
Conditioned medium from adipocytes reduces the lapatinib-induced cell cycle blockade in tumor cells. **A)** Lapatinib-induced cell cycle blockade was investigated on SKBR-3 cells incubated in control medium (a) or in adipocyte-conditioned medium (#3T3-CM) (b). Cells were exposed for 24 h to lapatinib before staining by propidium iodide and FACS analyses were performed to evaluate the percentage of cells in the different cell cycle phases. *n* ≥ 3. **B)** The expression of genes involved in the cell cycle progression was measured on tumor cells after exposure to lapatinib in presence (+) or not (−) of #3T3-CM. Cells were exposed for 12 h to lapatinib before RNA extraction and mRNA measurement by SYBR Green device. Fold changes were normalized for the cells exposed to lapatinib on the cells in the same condition not exposed to lapatinib. n ≥ 3

To assess lapatinib-induced cell cycle blockade, we stained the SKBR3 cells with propidium iodide and performed flow cytometry analyses of cells cultured in control medium or in #3T3-CM in the presence or absence of lapatinib. Figure 1a shows that the percentage of cells in G0/G1 phase was increased by 23.4% after exposure to lapatinib when SKBR3 were in control medium. The increase was lower for cells incubated in #3T3-CM (13.2%). The percentage of cells in S phase decreased from 14.3 to 5.8% when cells were incubated in control medium after lapatinib exposure whereas it decreased from 17.4 to 14.7% when incubated in #3T3-CM. The proportions of cells in G2/M phase followed the trend with a lower lapatinib-induced decrease for the tumor cells exposed to #3T3-CM than in control medium.

The results obtained when cells were incubated in control medium are consistent with the classical effect of lapatinib. However, this effect was weaker when SKBR3 cells were incubated in #3T3-CM, suggesting that lapatinib is less effective under these conditions. Interestingly, #3T3-CM had no effect on the proportion of cells in S phase as described in other studies [20].

After extraction and purification of cellular RNA, we performed quantitative RT-PCR assays to evaluate the transcription of genes coding for P27, cyclin D1, AKT and E2F3 (Fig. 1b). We normalized the fold changes obtained from three experiments for each condition with lapatinib on the cells in the same condition without lapatinib in order to highlight the transcription changes induced by the drug. As expected, in control medium, the exposure to lapatinib increased the expression levels of P27 and decreased those of AKT, cyclin D1 and E2F3 in tumor cells while the CM had no effect on its own (data not shown). For the cells exposed to #3T3-CM, P27 gene expression levels did not vary after exposure to lapatinib and those of cyclin D1 and E2F3 were higher than in cells cultured in control medium. The levels of AKT mRNA remained similar within all conditions.

### Adipocyte-conditioned medium induces a resistance in HER2+ breast cancer cells to several tyrosine kinase inhibitors

To evaluate and quantify the role of adipocytes in the sensitivity of tumor cells to lapatinib, we performed MTT assays and evaluated the drug-mediated cytotoxicity on several breast cancer cell lines with different levels of expression of HER2 in the presence of #3T3-CM or control medium.

**Fig. 2:**
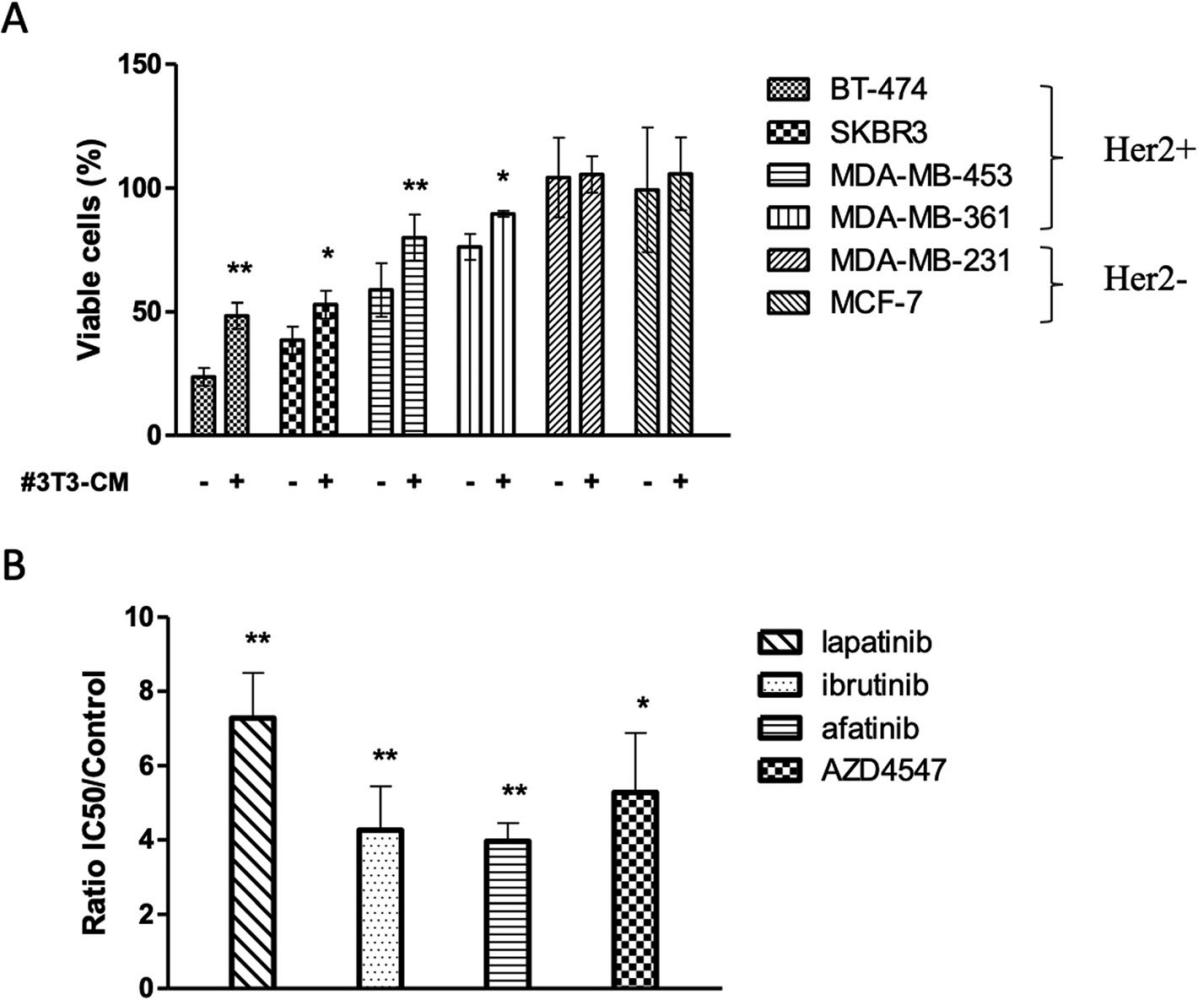
Adipocyte-conditioned medium induces a tumor cell resistance to various tyrosine kinase inhibitors in Her2 positive cell lines. **A)** BT-474, SKBR3, MDA-MB-453, MDA-MB-361, MDA-MB-231 and MCF-7 tumor cell lines were incubated 24 h in adipocyte-conditioned medium (#3T3-CM) or in control medium then we added 0,1 μM of lapatinib and we measured the mitochondrial metabolic activity by MTT that we correlated with the percentage of viable cells. *n* ≥ 3. *P* values were calculated by comparing for each cell line the percentage of viable cells in presence of #3T3-CM to the percentage of viable cells in control medium after exposure to lapatinib. **B)** Under the same conditions of incubation as in **A**) BT-474 cells were exposed to tyrosine kinase inhibitors lapatinib, ibrutinib, afatinib and AZD4547. n ≥ 3. The IC50 values for each therapeutic agent were measured and we calculated the ratio and evaluated the *p* values of the value in presence of #3T3-CM to the control media condition. *p < 0,05 **p < 0,01

The sensitivity of each cell line to lapatinib was quantified by calculating the mitochondrial metabolic activity that we correlated with the percentage of viable cells after exposure to lapatinib at 1 μM on each cell line (Fig. 2a). Lapatinib had a cytotoxic effect on all the HER2+ (BT-474, SKBR3, MDA-MB-453 and MDA-MB-361) cell lines but not on HER2- cells (MDA-MB-231 and MCF-7). However, this effect was significantly reduced when the HER2+ cells were cultured in #3T3-CM. These results show that #3T3-CM can induce resistance to lapatinib-induced cytotoxicity in HER2+ breast cancer cells. Moreover, the protective effect was found in both BT-474 cells which are HER2+/ER+/PR+ and SKBR3 cells, which are HER2+/ER−/PR-, suggesting that the effect is independent from estrogen and progesterone levels.

Lapatinib is not completely HER2-specific since it also binds to EGFR. In order to investigate whether #3T3-CM-induced resistance of tumor cells to lapatinib was specific of one or another TKR blockade, we performed MTT experiments to determine the half-maximal concentration (IC50) of other TKIs on BT474 cells. Ibrutinib is a TKI directed against Bruton tyrosine kinase but also against HER2, EGFR and HER3 [35]. Afatinib blocks HER2, EGFR and HER4 [36] and AZD4547 is an anti-fibroblast growth factor receptor (FGFR) [37]. Ratios were calculated using IC50 values obtained were cells were exposed to #3T3-CM over the IC50 values of cells in control medium for each drug and for each experiment (Fig. 2b). Quality controls for cytotoxicity assays included OD values greater than 0.2. All assays were performed with technical triplicates in at least three separate experiments.

The results revealed that the IC50 values were increased approximately two- to seven-fold for lapatinib when tumor cells were in contact of #3T3-CM in comparison to control medium. IC50s of ibrutinib were three and four-fold higher in #3T3-CM than in control medium. The exposure to #3T3-CM increased the IC50 of afatinib four-fold compared to the IC50 obtained in control medium. Finally, the IC50 for AZD4547 increased approximately five-fold for the BT-474 cells incubated in the #3T3-CM. Thus, the protection induced by exposure to adipocyte-CM was observed for every TKI tested.

### Exposure to adipocyte-derived factors is required during exposure to lapatinib to induce resistance

**Fig. 3:**
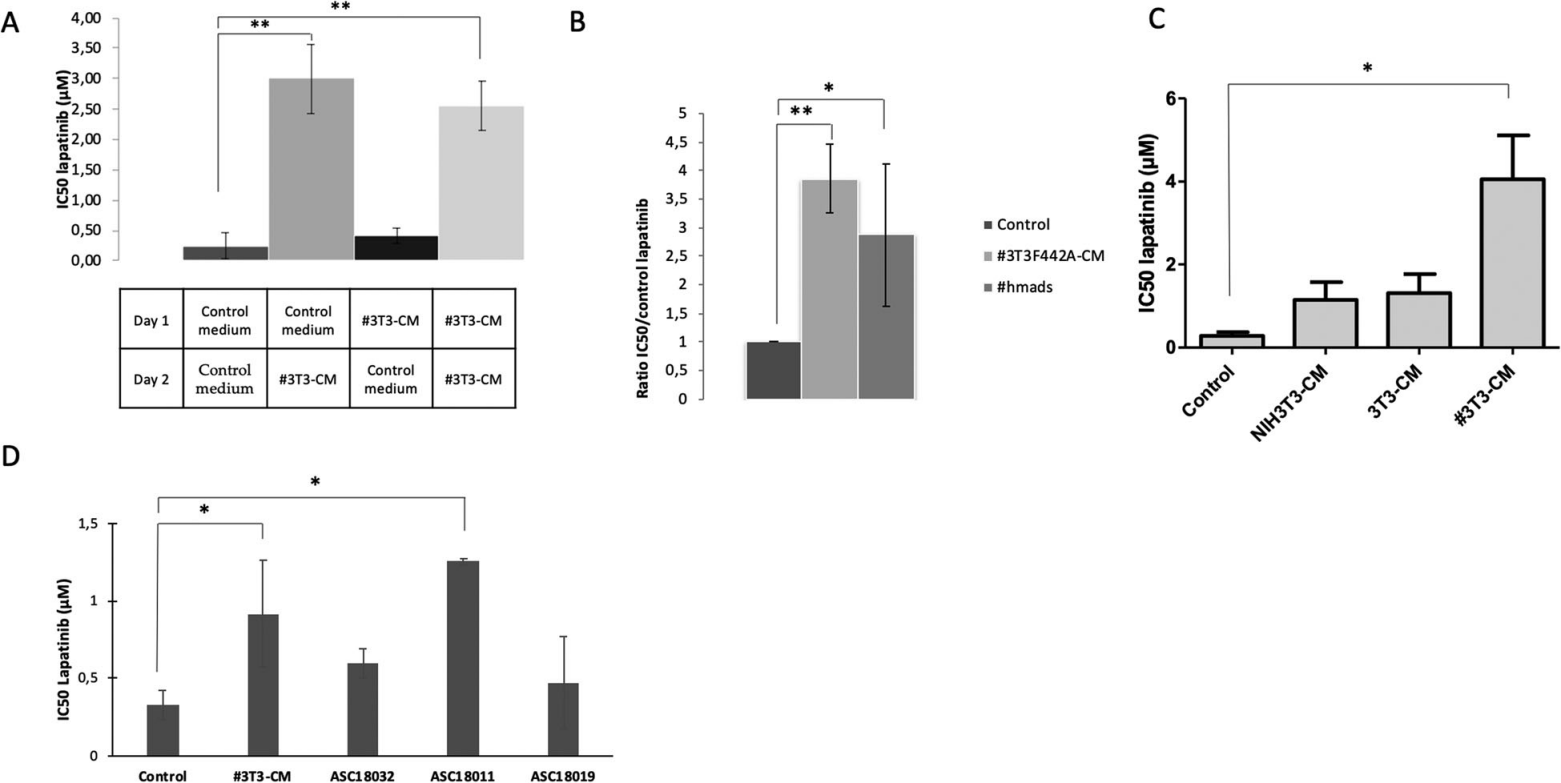
Adipocyte-conditioned medium-induced resistance of tumor cells to lapatinib is specific to adipocyte cell lines and the constant presence of adipocyte-conditioned medium is necessary to observe the resistance. **A)** BT-474 cells were seeded on the day 1 in control medium or adipocyte-conditioned medium (#3T3-CM) and the medium were changed on day 2 prior exposure to lapatinib and evaluation of IC50. n ≥ 3. **B, C** and **D** BT-474 cells were cultured in #3T3CM, in human adipocyte-conditioned medium (#hmads), in fibroblast-conditioned medium (NIH-3T3-CM), in preadipocyte-conditioned medium (3T3F442A-CM) or in conditioned medium from fresh human adipocytes from three donors (ASC18032, ASC15011 and ASC18019). We evaluated the IC50 of lapatinib in each condition. n ≥ 3. The ratio of IC50 for cells in control medium to the IC50 for cells in the various conditions. * *p* < 0,05 ** *p* < 0,01

In order to investigate whether the #3T3-CM-induced resistance of tumor cells to lapatinib required the presence of one (or several) soluble agent(s) during the exposure to lapatinib, we changed the conditions of #3T3-CM exposure (Fig. 3a). Cells were cultured (#3T3-CM or control medium) for 24 h and the medium was removed and replaced by new medium (#3T3-CM or control medium) shortly before the exposure to lapatinib. On the one hand, it appears that the BT-474 cells exposed for 24 h to #3T3-CM then placed in control DMEM medium prior to lapatinib exposure were as sensitive to lapatinib as the cells that had not been exposed to #3T3-CM. On the other hand, we observed a reduced sensitivity of tumor cells to lapatinib-induced cytotoxicity when these were first placed in control medium then secondarily exposed to the #3T3-CM. These results suggest that exposure to adipocyte-derived factors is required concomitantly to exposure to lapatinib to induce resistance to this agent.

### The protective effect is specific of adipocyte-conditioned medium

To confirm that the protective effect of adipocyte-conditioned medium could be observed with another adipocyte cell line we used the hMAD human adipocyte cell line. Using these cells, we also observed resistance of tumor cells to lapatinib in presence of hMAD-CM (Fig. 3b). Conversely in the presence of a non-adipocyte fibroblast-CM no resistance was observed, showing that the conditioned-medium from adipocytes specifically inhibits the activity of lapatinib on breast tumor cells (Fig. 3c). Additionally, #3T3-CM did not stimulate cell proliferation (supplementary Figure 1). We also incubated the tumor cells in fresh CM of human adipocytes obtained from three lean healthy patients (Fig. 3d). There was a trend towards higher IC50 values of lapatinib on cells exposed to these fresh adipocyte conditioned supernatants although the difference was only significant in patient ASC18011.

### Proximity with adipose tissue protects HER2+ cancer cells from lapatinib in vivo

**Fig. 4:**
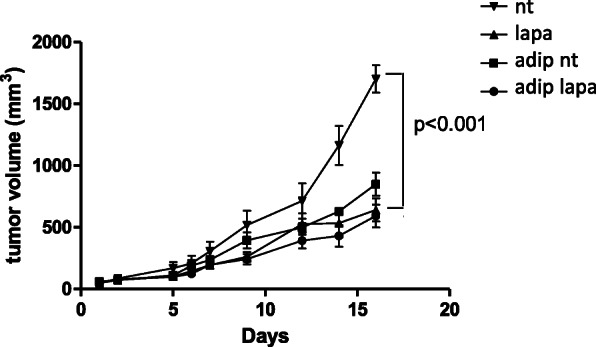
Proximity of adipose tissue protects tumors from the lapatinib-induced cytotoxicity in vivo. Tumor growth of BT-474 breast tumors in mice treated with 135 mg/kg of lapatinib daily were measured in four groups of five SCID mice. The animals of two groups were implanted with human adipose tissue (adip) 1 week prior to tumor xenografts. Animals were either treated with lapatinib (lapa) or not treated (nt)

To verify whether adipose tissue could exert a protective effect in vivo, we used a new and original adipose tissue xenograft model in SCID mice, which we recently reported [16]. Subcutaneous adipose tissue obtained from patients undergoing plastic surgery was injected subcutaneously into SCID mice to form a stable xenograft. BT-474 tumor fragments were secondarily grafted in contact with the adipose xenograft, to reproduce the contact between tumor and adipose tissue (Fig. 4).

Our results showed that lapatinib treatment significantly reduced BT-474 tumor growth in mice (lapa group) in comparison to the untreated group (nt group) (*p* = 0.005). Indeed, the tumor volume was 1700 ± 112 mm^3^ for the mice in the nt group and 600 ± 95 mm^3^ for those in the lapa group after 19 days, demonstrating the efficacy of lapatinib on the reduction of breast tumor growth. Conversely, lapatinib had no significant effect on tumors implanted in contact with adipose tissue xenografts (adip lapa group vs adip group). However, the tumor growth seemed to be impaired when the tumor was in contact with adipose tissue, a phenomenon that we have also observed with other models (data not shown). These results suggest that tumors in direct contact with adipose tissue are not sensitive to lapatinib.

### Soluble products issued from adipocyte lipolysis are likely to be responsible for the reduced antitumor activity of lapatinib on breast tumor cells exposed to adipocyte-conditioned medium

**Fig. 5:**
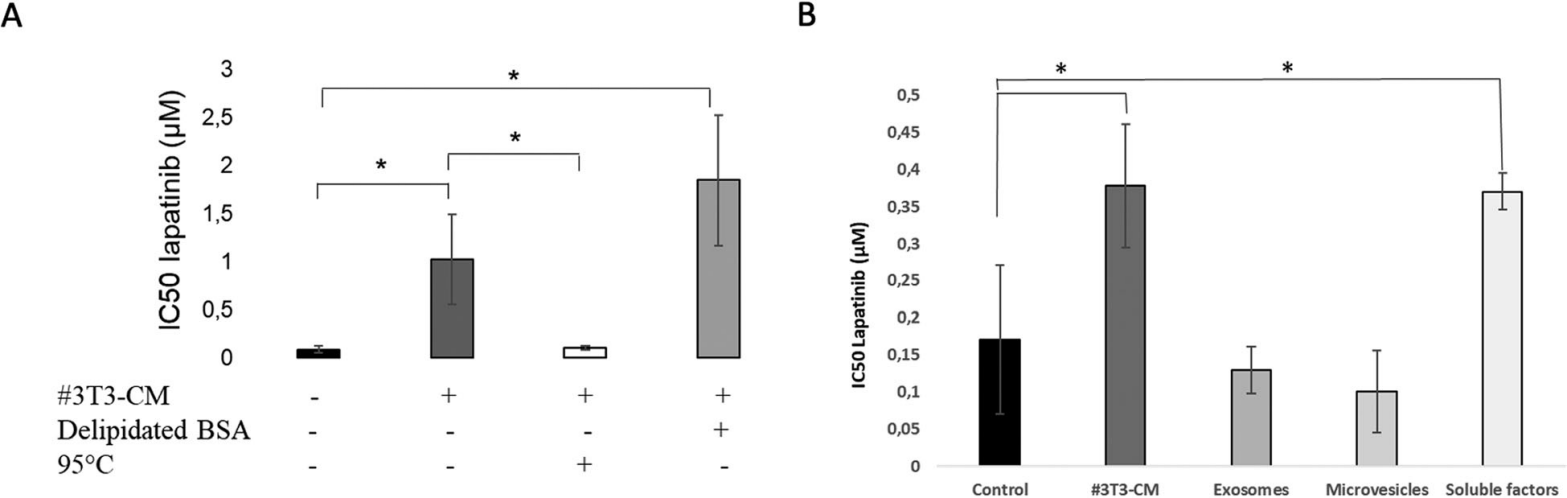
Thermolabile soluble factor(s) are responsible for adipocyte-conditioned medium-induced resistance of breast cancer cells to lapatinib. **A)** adipocyte-conditioned medium (#3T3-CM) were heated at 95 °C in order to denature the protein and delipidated BSA was added to the CM prior to exposure of tumor cells and to lapatinib. n ≥ 3. **B)** #3T3-CM were centrifuged to separate the microvesicles then ultracentrifuged to precipitate the exosomes and to isolate soluble factors. n ≥ 3. *P* values were calculated by comparing the conditions to the control medium. * *p* < 0,05

Due to their metabolism and endocrine functions, adipocytes produce a large variety of molecules including proteins and lipolytic products and release them as soluble molecules or contained in microparticles such as exosomes. In an attempt to identify the type of molecules responsible for the resistance effect induced to tumor cells, we performed different physical and chemical treatments on #3T3-CM. We heated the #3T3-CM at 95 °C in order to degrade the proteins, including adipokines and we added lipid-free BSA to sequester the lipids. We also isolated the exosomes by differential centrifugation and ultracentrifugation. Results shown in Fig. 5a suggest that the resistance of breast cancer cells to the effect of lapatinib depends on a thermolabile factor but not on free fatty acids. Indeed after #3T3-CM heating, we no longer observed the increase of lapatinib’s IC50 in breast tumor cells exposed to #3T3-CM compared to the same cells exposed to control medium, suggesting that thermolabile factor(s) had been degraded. Conversely, when we attempted to sequester the lipids by adding BSA and filtered the #3T3-CM, the protective effect of CM was maintained. After ultracentrifugation, we found that the #3T3-CM containing only the exosomes did not induced resistance to lapatinib-mediated while the #3T3-CM containing the soluble factors induced resistance to lapatinib (Fig. 5b).

**Fig. 6:**
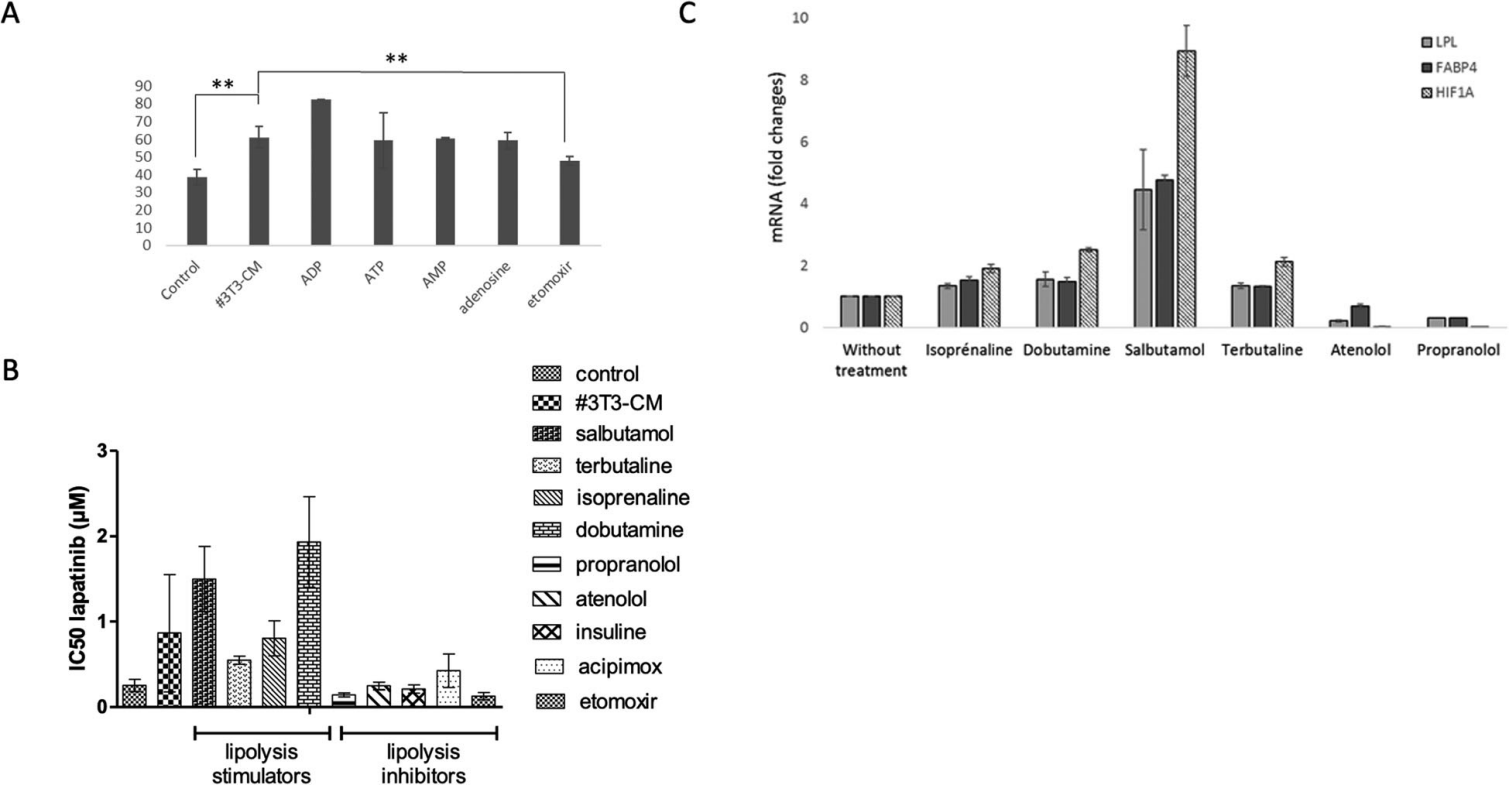
Inhibition of lipolysis in adipocytes reverses adipocyte-conditioned medium-induced resistance of tumor cells to lapatinib. **A)** Adipocytes were exposed to different metabolic modulators. After 6 h, the supernatants were harvested and added to BT-474 cells prior to exposure to lapatinib. BT-474 were also exposed to control medium or adipocyte-conditioned medium (#3T3-CM). n ≥ 3. **B)** adipocytes were exposed to lipolysis stimulators or inhibitors. The supernatants were harvested and used as in **A**) and IC50 values were calculated for each condition. n ≥ 3. **C)** mRNA was extracted from adipocytes after exposure to terbutaline, salbutamol, dobutamine, isoprenaline, atenolol and propranolol and the expressions of the genes involved in lipolysis stimulation were evaluated and normalized on the expression of the genes in adipocytes cultured without treatment. n ≥ 3. ** *p* < 0,01

As the secretome of adipocytes is very complex, we also attempted to pharmacologically modulate the metabolism of adipocytes in order to modify the adipocyte secretome of factors released from metabolic reactions. At first, as the metabolism of adipocytes is highly dependent on adenosine AMP, ADP and ATP [38, 39] we incubated the adipocytes with one or the other of these metabolites or with other molecule affecting lipolysis such as etomoxir that is a lipolysis inhibitor (Fig. 6a). We observed that the #3T3-CM from adipocytes exposed to agents such as ADP, AMP and adenosine maintained their ability to reduce the sensitivity of cancer cells to lapatinib. However, in the case of the CM from adipocytes exposed to etomoxir, we found a significantly higher reduction of viable tumor cells after exposure to lapatinib suggesting that the exposure to etomoxir could modify the production or release of soluble agent(s) responsible for the resistance phenotype provided by the CM.

As etomoxir modulates lipolysis by inhibiting the transport of fatty acyl chain from the cytosol into the mitochondria, we tried to verify our hypothesis that a soluble agent from lipolysis was responsible for the observed resistance by modulating the lipolysis via other pathways. We stimulated or blocked the lipolysis via β-adrenergic receptors. Indeed, the stimulation of a β-adrenergic receptor in adipocytes results in the increase of HIF1α transcription factor [40] and leads to the activation of lipolysis. We also investigated whether the effect of another lipolysis inhibitor, acipimox, an anti-lipolytic nicotinic acid and insulin derivative that is known to block lipolysis and to stimulate lipogenesis after food intake. As shown in Fig. 6b, we found that the #3T3-CM from adipocytes exposed to the antagonists of beta 1 and 2 adrenergic receptors called propranolol and atenolol did not induce resistance of BT-474 cells to lapatinib. This could be explained by the fact that these beta blockers are only effective when there this a beta-agonist present. Similar observations were made with other lipolysis inhibitors such as insulin and acipimox. Conversely, the exposure of adipocytes to pure or mixed beta 1 and beta 2 adrenergic receptor agonists such as salbutamol, terbutaline, isoprenaline and dobutamine produced a #3T3-CM that induced a resistance of tumor cells to lapatinib-mediated cytotoxicity at least as strong as #3T3-CM from adipocytes alone. To confirm that these agonists and antagonists were effectively modulating the receptors and altering lipolytic activities in adipocytes, we performed RT-PCR analysis of HIF1α as well as LPL and FABP4, the latter being involved in lipolysis (Fig. 6c). The results revealed a two to eight-fold increase in HIF1α expression in adipocytes exposed to the agonists compared to the unexposed adipocytes. LPL and FABP4 expression were also higher, particularly in adipocytes exposed to salbutamol. Conversely, the exposure to beta-blockers leads to a drop of the levels of mRNA for these three genes. These data are consistent with the hypothesis that adipose tissue induces a resistance to lapatinib of tumor cells via the release of lipolytic products in the microenvironment.

## Discussion

Adipose tissue and breast tumors are in close contact and adipocytes actively participate in tumor progression through the secretion of various adipokines [41]. Previous studies have shown that adipocyte-secreted factors could promote tumor cell resistance to various chemotherapeutic agents as well as antibodies. A similar effect has been demonstrated for multiple myeloma cells in response to melphalan, bortezomib, dexamethasone and doxorubicin [20, 32] and for breast cancer cells in response to trastuzumab, T-DM1 and gemcitabine [16, 33]. It has been found by Brady et al. that blocking mTOR could reverse lapatinib resistance of cancer cells [42]. Moreover, another study demonstrated that the activation of mTOR could elude HER2 blockade [43]. To our knowledge, no study has investigated the role of adipocyte-CM in breast cancer cell resistance to lapatinib or other TKIs.

Our results show that adipocyte-CM reduces the sensitivity of HER2+ breast tumor cells to the cytotoxic activity of lapatinib as well as other TKIs. These results have been confirmed in two different HER2+ cell lines and using conditioned medium from two different types of adipocytic cell lines. They are also in accordance with other studies using a CM from adipocytes demonstrating an adipocyte-induced resistance of tumor cells towards therapeutic agents. In our results, the cells exposed to #3T3-CM did not proliferate differently from cells in control medium. This suggests that the protective effect is not attributable to a reduced growth rate of cells due to adipocyte-derived factors. For the first time we also show that proximity to adipose tissue in vivo reduces sensitivity to lapatinib of established tumors. This confirms the relevance of adipocyte-derived factors in the in vivo setting.

Our results show a reduced lapatinib-induced cell cycle blockade when tumor cells are exposed to adipocyte-derived factors. This was observed both by direct analysis of the cell cycle and the expression of genes coding for the proteins involved in cell cycle progression. This type of resistance has already been described in the literature for chemotherapeutic agents [20–22]. Additional experiments could be performed to determine lapatinib-mediated reduction of expression of phosphorylated proteins in tumor cells such as HER2, EGFR, AKT, ERK1/2 in the presence or absence of adipocyte conditioned medium. Such results would highlight the role of downstream signaling pathways in the adipocyte secreted factor-mediated resistance of breast cancer cells to lapatinib.

Our results obtained using #3T3-CM fractionation and pharmacological modulation suggest that lipolysis pathway products are involved in the protective effect towards lapatinib. Modulation of lipolysis in differentiated adipocytes by etomoxir and acipimox, which act at two different levels of the metabolic reaction, were found to reduce #3T3-CM mediated resistance of breast tumor cells to lapatinib [44, 45]. An opposite effect was observed with beta-adrenergic receptor agonists. Additional metabolomic approaches are required to determine the soluble factor(s) involved in the resistance of tumor cells to lapatinib by measuring the metabolites in the #3T3-CM from adipocytes after exposure to the modulators of lipolysis. Such analyses have already been performed in supernatants from adipocytes [46].

Several mechanisms regarding resistance of tumor cells to lapatinib have been described in the literature [47]. An alternative hypothesis is that lapatinib could be sequestered or inactivated by adipocyte-derived soluble factor(s). Another mechanism of resistance could be the activation of compensatory pathway such as the increase activity of hepatocyte growth factor receptor (HGFR) or c-Met. The ligand of HGFR is the hepatocyte growth factor which is secreted by adipocytes. Other adipocyte-secreted factors have been described to promote tumor cell resistance to TKI, including the tissue inhibitor metalloproteinase-1 (TIMP-1), interleukin-6 (IL-6), the fibroblast growth factor (FGF) and neuregulin-1 (NRG-1) [48–52]. All these factors could play a role in the adipocyte-secreted factor mediated resistance of cancer cells to lapatinib observed in this study and it would be useful to quantify them or to evaluate their activity.

A possible limitation of our study includes the fact that we have used a murine adipocytic cell line and human breast cancer lines. Additional studies using a human pre-adipocytic line would be of interest. Another caveat is that we have explored the effect of proximal, rather than distal, adipose tissue on sensitivity to therapy. The use of obese mice models would constitute a means to address this issue. Finally, we have analyzed a limited number of kinase inhibitors in our models. A more extensive screening of kinase inhibitors with different affinities for various enzymes would be informative.

## Conclusions

The investigation of lipid metabolic reprogramming in the tumor microenvironment suggests a major role of cancer associated adipocytes [53, 54]. By using in vitro and in vivo approaches, we demonstrated that peritumoral adipocytes are likely to be involved in the resistance of breast tumor cells to lapatinib-mediated cytotoxicity. The effects of adipose cells are mediated via secreted factors that appear to affect HER2 associated downstream signaling pathways. This phenomenon is due to thermolabile soluble factors and is reversed when adipocytes are exposed to inhibitors of lipolysis. Further studies are necessary to identify the precise mechanism of this resistance and to allow the development of sensitizing strategies to circumvent adipose-mediated resistance to lapatinib in breast cancer.

## Supplementary information

**Additional file 1.**

## Data Availability

The datasets used and/or analysed during the current study are available from the corresponding author on reasonable request.

## Abbreviations

ADCC: Antibody-dependant cell-mediated cytotoxicity
AKT: Threonine kinase
CM: Conditionned medium
EGFR: Epidermal growth factor receptor
ER: Estrogen receptor
ERK1: Extracellular signal-regulated kinase-1
FABP4: Fatty acid binding protein 4
FGFR: Fibroblast growth factor receptor
HER: Human epidermal growth factor receptor
HER2: Human epidermal growth factor receptor-2
HER3: Human epidermal growth factor recpetor-3
HER4: Human epidermal growth factor receptor-4
HIF1α: Hypoxia Inducible Factor 1 Subunit Alpha
hMADS: human multipotent adipose derived stem cell
IL-6: Interleukin-6
LPL: Lipoprotein lipase
MAPK: Mitogen-activated protein kinase
mTOR: Mechanistic target of rampamycin kinase
MTT: 3-(4,5-dimethylthiazol-2-yl)-2,5-diphenyltetrazolium bromide
NRG-1: Neuregulin-1
P-gp: Permeability-glycoprotein
PI: Inorganic phosphate
PI3K: Phosphatidylinositol 3-kinase
PTEN: Phosphatase and tensin homolog
TIMP-1: Tissue inhibitor metalloproteinase-1
TKI: Tyrosine kinase inhibitor
TKR: Tyrosine kinase receptor
