# Supplementary material for: Adipocyte-conditioned medium induces resistance of breast cancer cells to lapatinib
Source: BMC Pharmacol Toxicol. 2020 Aug 14;21:61. doi: 10.1186/s40360-020-00436-z (PMC7427918; doi:10.1186/s40360-020-00436-z)
Supplement: Supplementary file 1 — Additional file 1. [file 40360_2020_436_MOESM1_ESM.pptx]

## Slide 1
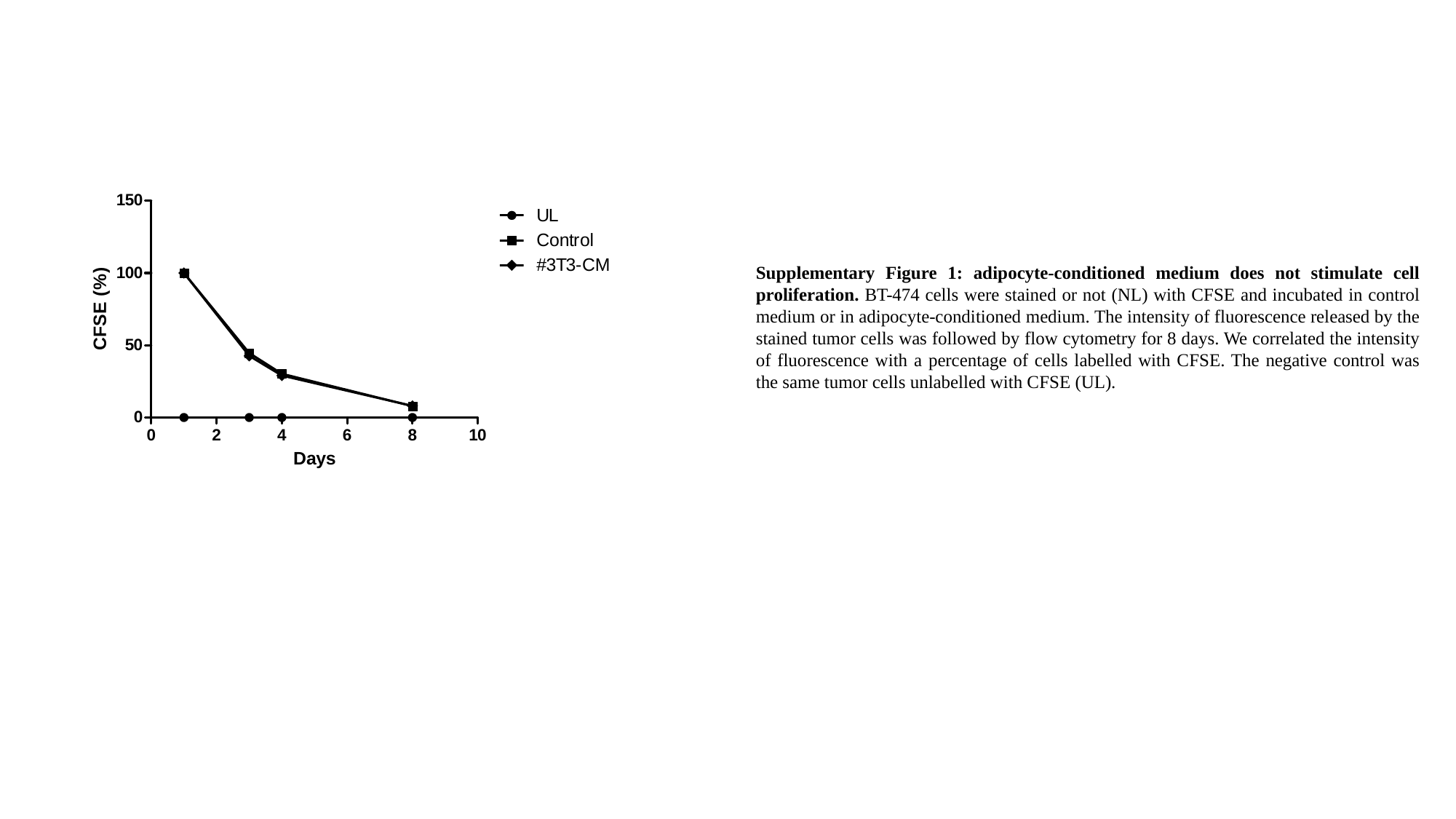

Supplementary Figure 1: adipocyte-conditioned medium does not stimulate cell proliferation. BT-474 cells were stained or not (NL) with CFSE and incubated in control medium or in adipocyte-conditioned medium. The intensity of fluorescence released by the stained tumor cells was followed by flow cytometry for 8 days. We correlated the intensity of fluorescence with a percentage of cells labelled with CFSE. The negative control was the same tumor cells unlabelled with CFSE (UL).
